# Summary of the Transactions of the College of Physicians of Philadelphia

**Published:** 1846-01

**Authors:** 


*Summary of the Transactions of the College of Physicians of Philadelphia*—From May to October, 1845, inclusive.

In this number of the semi-annual summary of the Transactions of this respectable body we find several subjects of interest discussed, and a number of valuable reports of cases, and papers on various subjects. A letter from Dr. Hubbard, of Hallowell, Maine, giving the result of his experience in the use of turpeth mineral (sub-sulphate of mercury,) excited considerable discussion, and elicited some valuable opinions on the subject of croup. We quote a case cited by Dr. H., showing his mode of administration and the indications for its use, and a portion of his remarks:

"I was called, in the night of Feb. 5, 1845, to see Miss C., aged about twelve years. In the three preceding nights, at about the same hour, she had been seized with paroxysms, similar to the one in which I found her,—each one continuing several hours, each of progressively increased severity, and each leaving her able to be about, during the day, with respiration so easy as to excite no alarm, and only a slight croupy cough. I found her with breathing extremely quick, laborious, and stridulous, the chest heaving with convulsive throes, the countenance livid, the eyes wild and distracted, extreme jactitation, the pulse thread-like, vaccilating and too rapid to be counted, the extremities, up to the body, of an icy coldness,—in short, the patient appeared like one in the last agonies. Strong rubefacients were immediately applied to the whole surface, sinapisms to the spine and extremities, and heated blankets to the lower limbs. As soon as she could swallow,—for deglutition was at first extremely difficult,—five grains of the *turpeth mineral* were given at once, and followed by the free use of mustard whey; this not vomiting her, after fifteen minutes I gave her five grains more, immediately upon swallowing which, vomiting commenced, and continued smartly for more than an hour. During this operation, and for some hours after, a free use was made of a decoction of seneka, as a stimulant diaphoretic. Her respiration began to improve immediately on vomiting, and, at the end of about four hours, it was comparatively good; the skin open, natural warmth restored, and the circulation equalized. The patient was then put upon the use of the following powder:

℞ Pulv. ipecac. comp., ℈j.

Hydrarg. chlorid. mit., ℈j.

Camphor. pulv., grs. xij.

M. ft. Chart. No. vj: One to be taken every four hours; a free use to be made of the decoction of seneka in the intervals, and the bowels to be moved after twenty-four hours.

"The emetic did not produce purgation. No paroxysm of difficult breathing returned. In forty-eight hours, my attendance ceased. I know of no other emetic substance that would have effectually met such a case. Ipecac., sulphate of zinc, &c., would have proved too feeble at best. Tartarized antimony, would, I conceive, have been hazardous, from its nauseating and sedative effect, setting aside the uncertainty of its procuring emesis in any reasonable dose, and the almost certainty of its producing exhausting catharsis. I repeat, that in this form and condition of croup, my experience furnishes me with no substitute for the *turpeth mineral*, as an emetic.

"In the more inflammatory and less paroxysmal forms of this disease, with decided arterial excitement, and hot skin, from the commencement, tartarized antimony, as a nauseant and emetic, may be, and undoubtedly is, with certain limitations, preferable. Still, I must say, that in all stages and conditions of this disease, where the equalizing and revellent effect of emesis is alone desired, I have never regretted having used the turpeth mineral, in preference to all other emetics.

"It remains, then, only to say a few words upon the peculiarity of this substance as an emetic, and upon some of the objections urged against it. In the first place, the promptness and certainty of its operation belong to no other substance within my knowledge. It has seldom, if ever, failed to vomit efficiently, when administered in a proper dose, in any of the various conditions of the stomach, and of the system, in which I have given it. It usually acts in ten or fifteen minutes, and the dose should be repeated at those intervals, if the first fail, which rarely happens. In efficiency and revellent power, it is not, perhaps, quite equal to the tartarized antimony; it is, however, vastly superior, in these respects, to ipecac. or any other substance known to me. In safety, it is greatly superior to antimony. Its emetic operation usually continues from an hour to an hour and a half, accompanied and followed by none of the distressing nausea, prostration, and depletion of antimony; but, on the contrary, leaving the patient with the invigorated feeling arising from equalized warmth and circulation. In its emetic operation, it has seldom, never in my recollection, been accompanied or followed by catharsis. I have never known it to be violent, nor otherwise than entirely safe in its operation, although I have given it in much larger doses than are usually directed; nor have I ever seen salivation follow its use as an emetic. So safe do I consider it, that in urgent cases I have not hesitated to put my patient under its full emetic operation, two or three times within twenty-four hours: nor have I seen ill consequences result from such practice. I am inclined to think that the dose should be somewhat larger than is usually recommended. From two to three grains may be given to a child two years old, and repeated in ten or fifteen minutes, until emesis is produced. If the first dose fails, the second usually acts as soon as it touches the stomach.

"I have thus given you, as you requested, the result of my experience in the use of this article—an experience somewhat extensive, during the last ten years:—such as it is, it is at your disposal."

In the discussion following the reading of the communication, Dr. Morris mentioned powdered alum as having been proposed to him by Dr. Meigs, as a safe emetic in similar cases, and gave his testimony to its efficacy. Dr. Bell considered croup an inflammatory disease, and mistrusted the power of simple emetics, and preferred to place reliance on depletion and tartar emetic. The indiscriminate use of Coxe's Hive Syrup, and other preparations of tartar emetic, as a domestic remedy, was strongly condemned in the remarks of several other medical gentlemen.

The annual Report on the Theory and Practice of Medicine, by Dr. Jackson, formerly of Northumberland, forms a portion of this number, and contains much of interest to practitioners in the West and South on the subjects of Miasmata, Malignant Intermittents and Idiomiasmatic Fevers. Extracts from this Report we propose to give in a future number.

"Dr. Ashmead stated that he had recently made some experiments in the venereal wards of the Philadelphia Hospital, to test the curative powers of the tartarized antimony, in cases of gonorrhœa.

"Four patients, affected with gonorrhœa, were treated with tartarized antimony alone. They were placed under the use of one-thirty-second of a grain, in solution, every hour—the dose being gradually increased to one-eighth of a grain, at the same intervals. The disease, in every case, rapidly disappeared, under this treatment—no one lasting more than a week, while, in one of them, it was said by a gentleman in attendance, to have terminated in forty-eight hours.

"In two of the cases, the gonorrhœa was complicated with inflamed testicle,—the only application made to which, was a simple poultice of bread and water." [Ed.

